# Lumiflavin increases the sensitivity of ovarian cancer stem‐like cells to cisplatin by interfering with riboflavin

**DOI:** 10.1111/jcmm.14409

**Published:** 2019-06-11

**Authors:** Ruhui Yang, Zhe Wei, Songquan Wu

**Affiliations:** ^1^ Department of Pharmacology College of Medicine and Health, Lishui University Lishui China; ^2^ Department of Rehabilitation Medicine College of Medicine and Health, Lishui University Lishui China; ^3^ Department of Immunology College of Medicine and Health, Lishui University Lishui China

**Keywords:** cancer stem‐like cells, cisplatin, lumiflavin, ovarian cancer, riboflavin

## Abstract

Here, we used lumiflavin, an inhibitor of riboflavin, as a new potential therapeutic chemosensitizer to ovarian cancer stem‐like cells (CSCs). This study demonstrates that the enrichment of riboflavin in CSCs is an important cause of its resistance to chemotherapy. Lumiflavin can effectively reduce the riboflavin enrichment in CSCs and sensitize the effect of cisplatin Diamminedichloroplatinum (DDP) on CSCs. In this study, CSCs of human ovarian cancer cell lines HO8910 were separated using a magnetic bead (CD133+). We also show the overexpression of the mRNA and protein of riboflavin transporter 2 and the high content of riboflavin in CSCs compared to non‐CSCs (NON‐CSCs). Moreover, CSCs were less sensitive to DDP than NON‐CSCs, whereas, the synergistic effect of lumiflavin and DDP on CSCs was more sensitive than NON‐CSCs. Further research showed that lumiflavin had synergistic effects with DDP on CSCs in increasing mitochondrial function damage and apoptosis rates and decreasing clonic function. In addition, we found that the combination of DDP and lumiflavin therapy in vivo has a synergistic cytotoxic effect on an ovarian cancer nude mice model by enhancing the DNA‐damage response and increasing the apoptotic protein expression. Notably, the effect of lumiflavin is associated with reduced riboflavin concentration, and riboflavin could reverse the effect of DDP in vitro and in vivo. Accordingly, we conclude that lumiflavin interfered with the riboflavin metabolic pathways, resulting in a significant increase in tumour sensitivity to DDP therapy. Our study suggests that lumiflavin may be a novel treatment alternative for ovarian cancer and its recurrence.

## 1 INTRODUCTION

Ovarian cancer is among the most common malignant tumours in women. Because of its resistance to chemotherapy and its recurrence thereafter, its incidence is inferior to cervical and endometrial cancer, but its mortality ranks first among gynaecological malignant tumours.^1^ Cancer stem‐like cells (CSCs) are subsets of tumour cells that have the capacity of stem cells in tumour tissues and are important for the recurrence and chemotherapy resistance of tumour.^2^ Therefore, it is important to enhance the treatment strategies for CSCs to reduce the recurrence and resistance of ovarian cancer.

The ability of CSCs to resist chemotherapy injury is stronger than that of ordinary tumour cells.^3^ In this study, we speculate a close connection between riboflavin and the resistance of CSCs to chemotherapeutic agents. First, a unique phenomenon occurs, wherein CSCs can be enriched by riboflavin.^4^ Second, in the process of cancer treatment, cell apoptosis is induced by chemotherapeutic agents, involving excessive production of reactive oxygen species (ROS) and DNA damage.^5^ Antioxidants may accelerate the growth of early tumour progression in mice.^6^ Third, riboflavin, a coenzyme of some important oxidation reduction enzymes, participates in cell biological oxidation and increases cell anti‐oxidation. The insufficiency of riboflavin reduces the antioxidant capability of cells against ROS.^7^, ^8^ Moreover, the interference of riboflavin metabolism improves the sensitivity and reduces the adhesion and migration of ovarian cancer cells to Diamminedichloroplatinum (DDP). These effects are associated with the reduced percentage of stem cell‐like tumour cells.^9^, ^10^ Therefore, interfering with riboflavin metabolism may be a potential target for CSCs.

**Figure 1:**
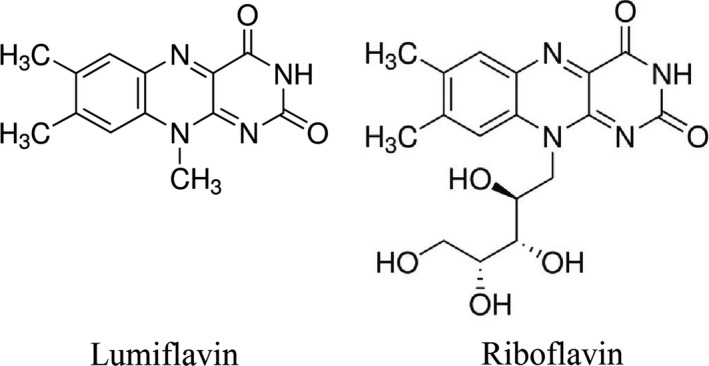
Chemical structure of lumiflavin and riboflavin

Lumiflavin, a natural inhibitor of riboflavin (Figure 1), can prevent the conversion of riboflavin into flavin adenine dinucleotide (FAD) and flavin mononucleotide, and can interfere with the physiological effect of riboflavin.^11^, ^12^ In this study, we explore the efficacy of lumiflavin, a riboflavin inhibitor, in both murine models and human cell lines in vivo and in vitro and found that it synergized with DDP therapy in ovarian cancer. Our results show that the key pathways, such as riboflavin metabolic pathway, are essential in the action maintenance of CSCs and DDP chemoresistance, suggesting an important clinical application of lumiflavin in ovarian cancer therapy.

## 2 MATERIAL AND METHODS

### 2.1 Cell line, drugs, animal, reagents and equipment

Human ovarian cancer cell lines HO8910 were purchased from Shanghai Cell Bank of Chinese Academy of Sciences; riboflavin was purchased from National Institutes for Food and Drug Control of China; lumiflavin was purchased from Toronto Research Chemicals (TRC) of Canada; Accuri C6 flow cytometry was made by BD Biosciences of USA; Real‐time Quantitative PCR Detecting System System and Western blot system were made by Bio‐Rad Laboratories of USA. Specific pathogen‐free CAnN.Cg‐Foxn1nu/Crl mouse (BALB/c) nude female mice with an age of approximately 6 weeks and weight of 18‐22 g were provided by Shanghai Slack Laboratory Animal Co., Ltd. (Shanghai, China). The animals were housed in a temperature‐controlled room with 12‐hour dark/light cycles and were given access to food and water ad libitum. This study was performed in strict accordance with the recommendations of the Guide for the Care and Use of Laboratory Animals of the National Institutes of Health. The protocol was approved by the Animal Research Ethics Board of the Lishui University (Lishui, Zhejiang Province, China, Permit Number: 0401‐2017).

### 2.2 Magnetic bead separation and flow cytometry

HO8910 cells were cultured in DMEM containing 10% foetal bovine serum (FBS) supplemented with 1 × 10^5^ IU/L each of penicillin and streptomycin at 37°C in 5% CO_2_ atmosphere. The medium was changed every 2‐3 days depending on cell growth. Cancer stem‐like cells were isolated through magnetic bead sorting by using a magnetic cell sorting system (MACS) based on a previous study.^13^, ^14^ Briefly, the cells were harvested from each of the HO8910 cell lines. According to the manufacturer's instructions, the cells were then labelled with CD133 antibodies conjugated to magnetic beads (Miltenyi Biotec) and then co‐labelled with CD133 conjugated to PE (Miltenyi Biotec). Antibody positive and negative cells were then separated using MACS‐LS separation columns (Miltenyi Biotec) and identified as antibody‐enriched or ‐depleted cell populations. CD133+ cells were maintained in ultralow attachment plates (Corning Costar Corporation, USA) in knockout DMEM/F12 medium supplemented with 20% knockout serum replacement (Life Technologies), 20 ng/mL epidermal growth factor, and 10 ng/mL basic fibroblast growth factor with 10 ng/mL leukaemia inhibitory factor. All cells were grown at 37°C in humidified atmosphere of 5% (v/v) CO_2_.^15^, ^16^ To detect the purity and character of CSCs (CD133+), the CD117 (Boster Biological Technology, China), CD133 (Miltenyi Biotec), CD44 (BD Biosciences) and CD24 antibodies (BD Biosciences) were subjected to flow cytometry (BD Biosciences, San Jose, CA) according to the manufacturer's instructions. The cells were centrifuged at 1500*g* for 5 minutes. The supernatant was discarded, and the cell pellet was re‐suspended in PBS and then ran on a flow cytometer. CD117‐FITC and CD24‐FITC were detected by the FL1A channel, while CD133‐PE and CD44‐PE were detected by FL2A channel. The data were analysed using the BD Diva Software to calculate the percentage of double positive.

### 2.3 Detection of the mRNA and protein expression of riboflavin transporter 2 in CSCs and non‐CSCs of HO8910

Reverse‐transcription polymerase chain reaction was used to determine the mRNA expression levels of riboflavin transporter 2 (*RFT2*) in CSCs and NON‐CSCs of HO8910. Total RNA was extracted from cells by using the TRIzol reagent (Invitrogen, USA), according to the manufacturer's instructions. An IQ SYBR Green SuperMix PCR array kit was purchased from Vazyme Biotech Co., Ltd. (China). Two micrograms of extracted RNA was converted into cDNA by using the MMLV‐reverse transcriptase (Fermentas, Canada), according to the manufacturer's instructions. The cDNA was amplified using the following forward and reverse primers as previously described 17, 18: forward: 5′‐CCTTTCC GAA GTGCCCATC‐3′ and reverse: 5′‐AGAAGGT GGTGAGGTAGTA GG‐3′; and β‐actin, forward: 5′‐AGCCAGACCGTCTCCTTG TA‐3′ and reverse: 5′‐TAGAGAG GGCC CACCACAC‐3′. The human β‐actin housekeeping gene was used as an internal control. The primers were designed and synthesized at Shanghai Generay Biotech (Shanghai, China). After carrying out the reaction, the results were analysed using the computational fluid dynamics X Connect RT‐PCR System. The relative expression level of the mRNA in each sample was calculated by normalizing its threshold cycle (Ct) value to the Ct value of β‐actin housekeeping gene by using the 2^−ΔΔCt^ method. These levels were expressed in arbitrary units. The RFT2 expression of HO8910 was detected using Western blot analysis. The total protein from the CSCs and NON‐CSCs was prepared under reducing conditions by using 4%‐12% Bis‐Tris SDS‐PAGE gels before being blotted and detected using anti‐RFT2 Rabbit antibody (ab223094; Abcam, USA). Protein expression analysis was performed with the Imagej 1.44 Quant software. The distribution of RFT2 in cells was detected using immunofluorescence method. Cancer stem‐like cells and NON‐CSCs were washed thrice by using PBS, and the cells were fixed using 4% paraformaldehyde for 30 minutes. The 0.1% Triton X‐100 solution was used to cover the cell surface, followed by permeabilization for 30 minutes and blocking with 5% bovine serum protein solution for 2 hours at 37°C. Primary antibody (ab223094; Abcam) was added after rinsing the cells thrice by using PBS, followed by incubation overnight at 4°C. Diluted fluorescent secondary antibody was added after rinsing the cells thrice by using PBS, followed by incubation for 60 minutes at 37°C in the dark. After 4',6‐diamidino‐2‐phenylindole (DAPI)‐staining of the nuclei, the expression of RFT2 (red fluorescence) was detected through laser confocal microscopy. Imagej 1.44e professional image analyzer was used to analyse the intensity of the fluorescent expression.^18^

### 2.4 The contents of riboflavin in cells and tissues were measured by ultra‐high performance liquid chromatography‐tandem mass spectrometry

Riboflavin was detected in cells and tissues through ultra‐high performance liquid chromatography‐tandem mass spectrometry (UPLC‐MS/MS) as described previously.^19^ Briefly, UPLC‐MS/MS was carried out using a Waters ACQUITY I‐Class UPLC system (Waters Corp., Milford, MA), and the chromatographic separation was performed on the C18 column (2.1 mm × 50 mm, 1.6 μm; Waters Corp.) at a column temperature of 40°C. The mobile phase consisted of acetonitrile (A) and 0.1% formic acid in water (B). The column was eluted with a linear gradient of A at 10% for 0‐0.3 minutes and 10%‐95% for 0.3‐0.7 minutes. The composition was held at 95% A for 1.1 minute, and then returned to 10% A. The total run time for the analytes was 3 minutes. The flow rate was set at 0.40 mL/min at an injection volume of 2 μL. Ultra‐high performance liquid chromatography‐tandem mass spectrometry was conducted on a Benchtop tandem quadrupole mass detector designed for ultra high performance triple quadrupole mass spectrometer (Waters Corp.), equipped with electrospray ionization interface. Nitrogen was used as the desolvation gas and cone gas at a flow rate of 1000 and 50 L/h respectively. The ion monitoring conditions were set at a capillary voltage of 1 kV, source temperature of 150°C and desolvation temperature of 500°C. For the quantitative analysis, the multiple reaction monitoring mode with positive electrospray ionization of m/z377.1 → 243 was set for riboflavin and m/z 257 → 186 for lumiflavin.

### 2.5 Cell vitalities detected by CCK‐8 kit [Cell counting kit‐8, Sodium 4‐(3‐(2‐methoxy‐4‐nitrophenyl)‐2‐(4‐nitrophenyl)‐2H‐tetrazol‐3‐ium‐5‐yl) benzene‐1,3‐disulfonate (WST‐8)]

Cells were collected and the cell viability was determined using the CCK‐8 assay kit (Dojindo Laboratories, Japan) according to the manufacturer's instruction after 48 hours of treatment. The optical density (OD) value of each well was read at 450 nm. Each group had six‐well plates. Cell viability was calculated using the following formula: Cell viability (%) = (OD [experiment group − OD [blank])/(OD [control group] − OD [blank]) × 100.^20^

### 2.6 Cell mitochondrial membrane potential detected by flow cytometry

Cells were collected from each group 48 hours after treatment. A total of 1 × 10^5^ cells from all groups were digested, collected through centrifugation, suspended and mixed with a 5,5',6,6'‐tetrachloro‐1,1',3,3'‐tetraethylbenzimi ‐ dazolylcarbocyanine iodide (JC‐1) working liquid probe (Beyotime Institute of Biotechnology, Jiangsu, China) accordance to the manufacturer's protocol.^21^, ^22^, ^23^ The cells were incubated at 37°C for 20 minutes, and the centrifugal cells were washed twice with 4°C buffer. The mitochondrial membrane potential (Δ*ψ*_m_) was determined using the JC‐1 probe according to the manufacturer's instruction, and the data were analysed using an Accuri C6 flow cytometer. The stained cells were analysed using a flow cytometer equipped with the CellQuest software.

### 2.7 ROS level detected by flow cytometry

A total of 2 × 10^5^ cells from each group was collected 48 hours after drug treatment through centrifugation. The cells were suspended with serum‐free medium, and then incubated at 37°C for 20 minutes. The supernatants were removed after centrifugation, and the cells were washed thrice times by using the serum‐free medium. Finally, the ROS levels were detected using the 2,7‐Dichloro‐dihydrofluorescein Diacetate (DCFH‐DA) kit (Beyotime Institute of Biotechnology), according to the manufacturer's instructions. The ROS levels (10^4^ DCF‐H) were detected using an Accuri C6 flow cytometer equipped with the CellQuest software.^24^

### 2.8 Apoptosis assay by flow cytometry

A total of 1 × 10^5^ cells were collected through centrifugation 48 hours after the treatment. The cells were digested using 0.25% trypsin (excluding ethylenediaminetetraacetic acid), washed twice (centrifugation at 1000×g for 5 minutes) with 4°C pre‐cooled PBS, added with 5 μL of Annexin V‐FITC and 10 μL of propidium iodide (Multi Sciences Biotech Co., Ltd., Zhejiang, China), and then incubated at 4°C for 5 minutes. The rate of apoptosis was assayed through flow cytometry.^25^

### 2.9 Colony formation assay

The cells were re‐suspended in culture medium containing 10% FBS at a density of 2 × 10^3^/dish into a six‐hole plate. The cells were held for 2 weeks, and colony formation was monitored. To evaluate colony formation, cells were fixed and then stained with crystal violet (Beyotime Institute of Biotechnology) for 15 minutes after the culture period. The clones consisting of a minimum of 50 cells were counted.^26^

### 2.10 Mouse xenograft model

Female BALB/c nude mice (5‐6‐weeks‐old) were maintained in caged housing in a specifically designed pathogen‐free isolation facility with a 12/12 hour light/dark cycle. The CSCs of HO8910 (5 × 10^6^) cell line were subcutaneously injected under the oxter skin of nude mice. When the tumours reached a palpable size (100 mm^3^),^27^ the mice were randomly divided into six groups and intraperitoneally injected with saline control, riboflavin (4 mg/kg, once a day), lumiflavin (8 mg/kg, once a day), DDP (5 mg/kg, once a week), DDP + lumiflavin and DDP + riboflavin. All group mice were treated for 25 days. The tumour size was monitored twice a week, while tumour weight was measured at the end of the experiment.^18^ Tumour size was measured using the following formula: *V* = *L* × *W* × *D* × π/6, where *V* is the volume, *L* is the length, *W* is the width and *D* is the depth.^28^

### 2.11 Determination of Glutathione Peroxidase (GSH‐PX) and Superoxide Dismutase (SOD) enzyme activity, and MDA content in tumour tissue

The tumour tissues of mice were washed with cold saline, weighed and then made into 10% homogenate by adding normal saline. The homogenate was then centrifuged at 5000 *g* for 10 minutes at 40°C to prepare the supernatant. The activities of GSH‐PX and SOD and the MDA content were measured directly using the commercial kits (Nanjing Jiancheng Bioengineering Institute, Nanjing, China).^29^

### 2.12 Data analysis

All experiments were repeated at least three times and the data are presented as the mean ± S.D. Differences between data groups were evaluated for significance using the Student's *t* test of unpaired data (two‐tailed). For animal studies, the data are presented as the mean ± S.E.M. The *F* test for the homogeneity of variance was conducted. The tumour volume was analysed with one‐way ANOVA using the software SPSS 11.5 for Windows (Chicago, IL, USA). Significant and highly significant differences were considered at *P* < 0.05 and *P* < 0.01 respectively.

## 3 RESULTS

### 3.1 Differences observed between the NON‐CSCs and CSCs of HO8910 cell line in terms of riboflavin levels, sensitivity to DDP, mRNA and protein expression of RFT2, as well as the synergetic effect DDP combined with different dosages of lumiflavin

The CSCs of the HO8910 cell line were isolated through the CD133+ magnetic bead separation method. To detect the characteristics of CSCs, key stem‐cell surface markers, such as CD117+/CD133+, CD44+/CD177+ and CD44+/CD24− were analysed using a flow cytometer. The double positive cell of CD117+/CD133+ is 94.7%, CD44+/CD177+ is 88.8% and CD44+/CD24− is 71.9%, compared to that of HO8910 cell at 14.2%, 2% and 0.43% (Figure 1A,B). Furthermore, the mRNA and protein expression levels of RFT2 were tested using RT‐PCR, Western blot analysis and immunofluorescence technique. The results show that the relative gene expression of *RFT2* was more than seven times in CSCs (2.95 ± 0.48) than NON‐CSCs (0.76 ± 0.34), and that of RFT2 in CSCs (0.81 ± 0.14) was more than twice of than in NON‐CSCs (0.41 ± 0.04). And the results of immunofluorescence staining present the RFT2 expression of cell membranes, and the luminous density of CSCs was stronger than that of NON‐CSCs (Figure 1 C‐G). Besides, to detect the different distributions of riboflavin and RFT2 in CSCs and NON‐CSCs, UPLC‐MS/MS was conducted. The results show that the riboflavin content is 2203 ± 142 ng/g protein in CSCs, which is approximately thrice of that in NON‐CSCs (748 ± 21 ng/g protein) (Figure 1H). These results suggest that the mRNA and protein expression abundance of RFT2 is significantly higher in CSCs than in NON‐CSCs.

To test the difference of sensitivity to DDP between CSCs and NON‐CSCs, cells were seeded in 6‐well plates (1 × 10^6^ cells per well) and randomly divided into seven groups including the control group and DDP group at 5, 10, 20, 40 m and 80 μmol/L. The cells were collected from each group 48 hours after treatment. Cell vitalities were detected using the CCK‐8 kit. The results show that NON‐CSCs are more sensitive to DDP than CSCs. CSCs show resistance to chemotherapy unlike NON‐CSCs (Figure 1I).

The synergistic effect sensitivity of lumiflavin combined with DDP was tested on CSCs and NON‐CSCs. Two types of cells were treated with 10, 20, and 40 µmol/L of lumiflavin and 20 µmol/L of DDP for 48 hours. The results show that the synergistic effect of lumiflavin and DDP was significantly better on CSCs than on NON‐CSCs (Figure 1J).

### 3.2 Effects of lumiflavin combined with mitochondrial membrane potential of DDP, ROS, and apoptotic and colony‐forming ability on CSCs

**Figure 2:**
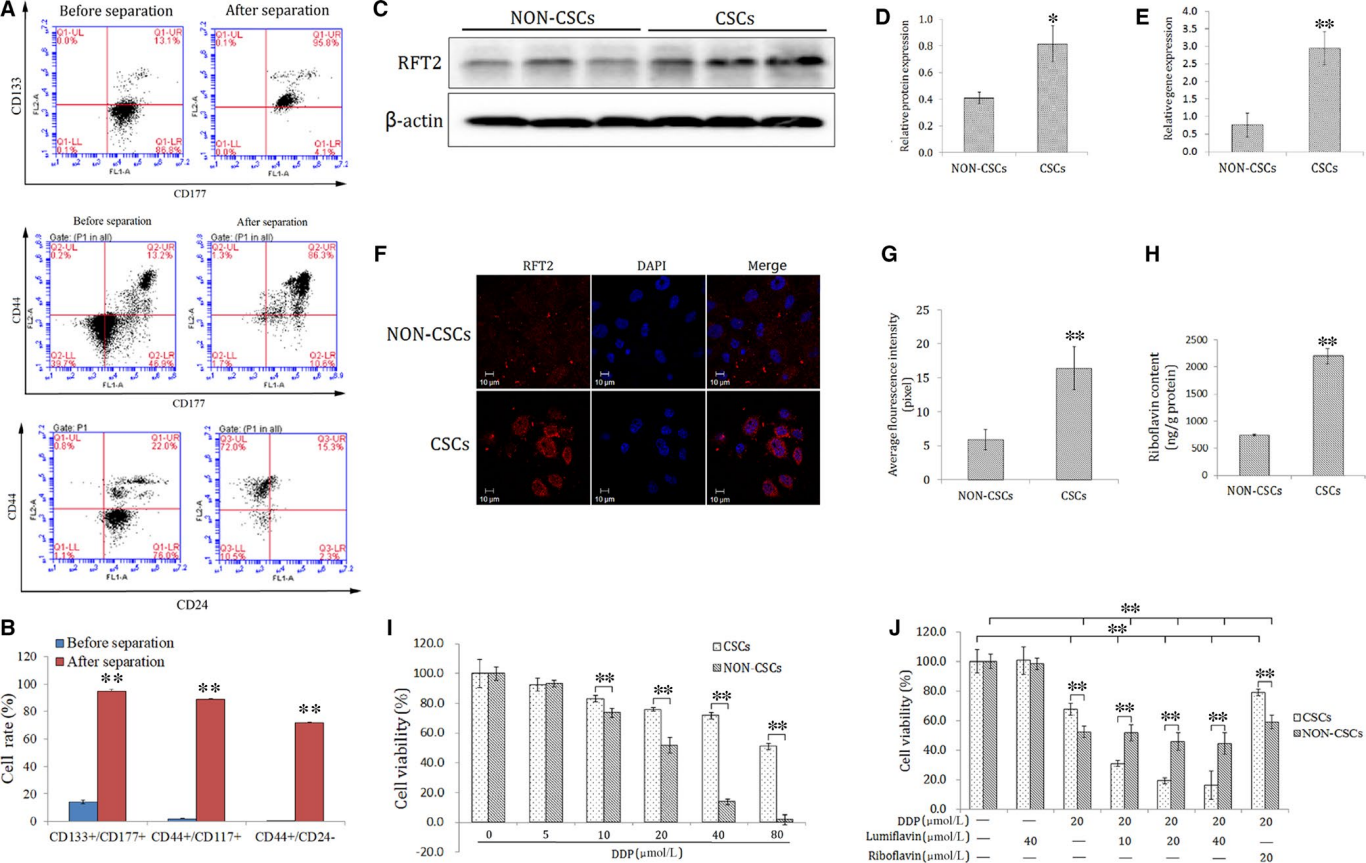
Difference in the cancer stem‐cell surface markers, riboflavin content, expression levels of riboflavin transporter 2 (*RFT2*) gene and protein, sensitivity to DDP, and the synergistic effect of lumiflavin and DDP between cancer stem‐like cells (CSCs) and NON‐CSCs. CSCs of human ovarian cancer cell lines HO8910 were isolated through magnetic bead sorting (CD133+). Co‐expression of CD133+/CD117+, CD44+/CD177, CD44+/CD24− were detected through flow cytometry, riboflavin contents were detected using ultra‐high performance liquid chromatography‐tandem mass spectrometry, and expression levels of the *RFT2* mRNA and protein were examined using RT‐PCR, Western blot analysis and immunofluorescence methods. CSCs and NON‐CSCs were treated with different concentrations of DDP and with lumiflavin combined with DDP for 48 h. Cell viability was detected using CCK‐8 methods. A, Flow detection results of CD133+/CD117+, CD44+/CD177, CD44+/CD24− cells ratio of ovarian cancer cell lines HO8910 before and after by magnetic bead (CD133+) separation. B, Statistical analysis graph of A (n = 3). C, Electrophoretograms of RFT2 proteins. D, Graph of relative protein expression as determined by Western blot analysis (n = 3). E, Statistics of the gene expression of *RFT2* as determined by RT‐PCR (n = 3). F, Immunofluorescent images of the RFT2 protein (red dots) and DAPI (blue dots) of CSCs and NON‐CSCs. G1‐G3: NON‐CSCs; G4‐G6: CSCs. G, Quantification of RFT2 dots per field. In total, 30 fields from three cell images were analysed per group. H, Statistical graph of riboflavin contents of CSCs and NON‐CSCs (*n* = 3). (I,J) Statistical maps of cell activities (n = 6). *P < 0.05 between groups; **P < 0.01 between groups. Mean ± SD

The synergistic effect of lumiflavin and DDP on CSCs was further explored. Mitochondrial membrane potential, ROS contents, and apoptotic and multiplication capacity were tested through flow cytometry and plate clone formation assay. The results show that lumiflavin could synergistically increase the effect of DDP on CSCs to increase mitochondrial depolarization ratio and ROS contents. Further experiments show that DDP treatment with lumiflavin could increase the cell apoptosis rate. In addition, the cloning results show that lumiflavin and DDP treatment decreased the colony‐forming ability, compare the DDP group. However, the effects DDP could be overturned by riboflavin. Treatment with either riboflavin or lumiflavin minimally affected CSCs (Figure 2A‐H).

### 3.3 Synergistic effect of lumiflavin and DDP on CSCs, and antagonistic effect of riboflavin on DDP in a xenograft model

**Figure 3:**
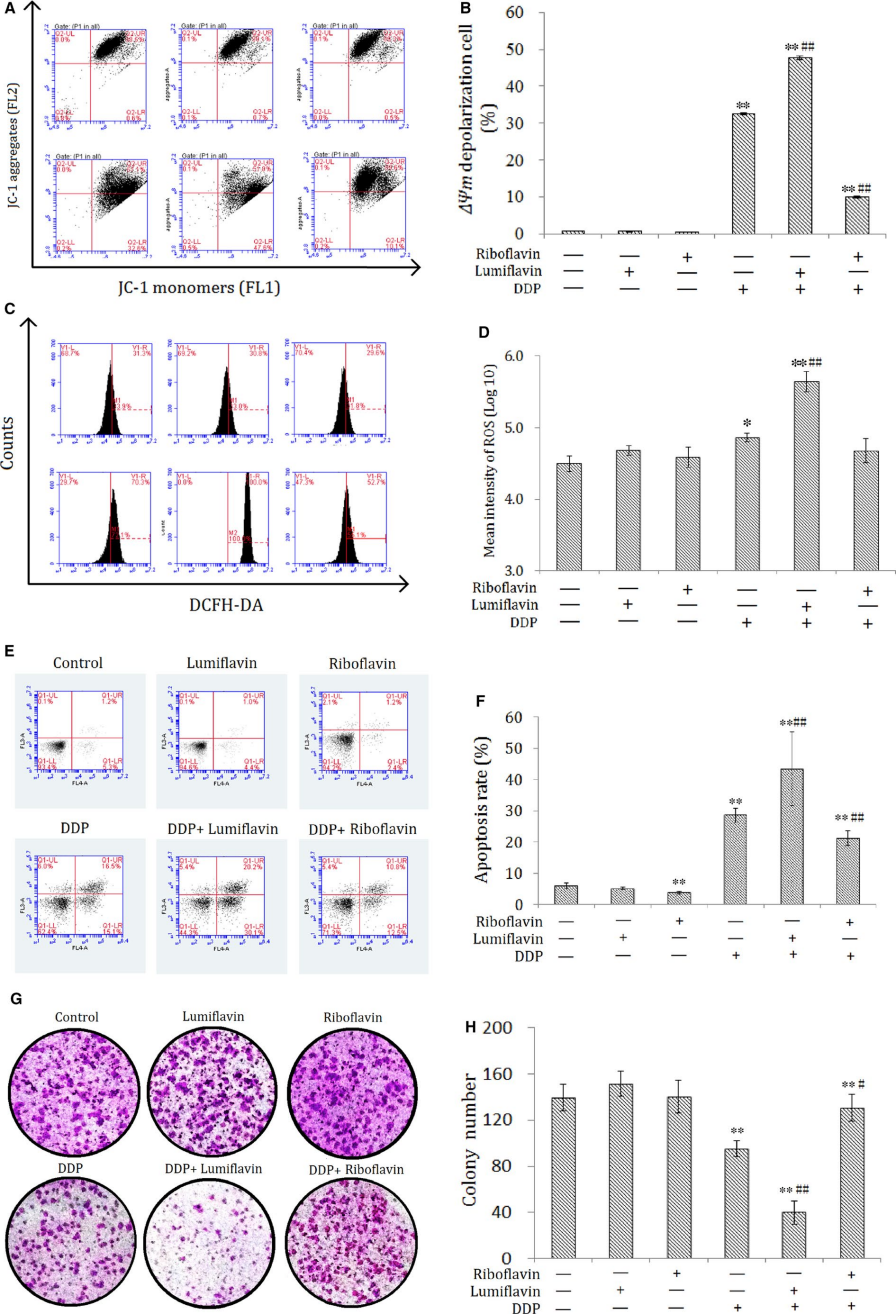
Synergistic effects of lumiflavin and DDP on mitochondrial membrane potential, reactive oxygen species (ROS) contents, apoptosis rate, and clonality of cancer stem‐like cells (CSCs). CSCs were seeded in 6‐well plates (1 × 10^6^ cells per well). Cells were randomly divided into the control group, lumiflavin (20 μmol/L) group, DDP (20 μmol/L) group, DDP + lumiflavin (20 μmol/L) group and DDP (20 μmol/L) + riboflavin (20 μmol/L) group. After treatment for 48 hours, the membrane potential, ROS contents and apoptosis rate were detected using a flow cytometer. CSCs were seeded in 6‐well plates (2 × 10^3^/well) and held for 2 weeks. The colony were fixed and then stained with crystal violet to evaluate the colony formation. A, Flow cytometry scatter plot of the potential of the mitochondrial membrane (Δ*ψ*_m_) of cells as detected through flow cytometry by using a JC‐1 probe. B, Statistical analysis graph of (A). C, Flow cytometry of ROS levels as detected using the DCFH‐DA kit. D, Statistical analysis graph of (C). E, Flow cytometry of cell apoptosis as detected using Annexin V‐FITC. F, Statistical analysis graph of (E). G, Images of colony formation assay. H, Statistical analysis graph of (G). *P < 0.05 compared to control group; **P < 0.01 compared to control group. ^#^ *P* < 0.05 compared to DDP group; ^##^ *P* < 0.01 compared to DDP group. Mean ± SD (n = 6)

To verify the synergistic effect of lumiflavin on ovarian cancer CSCs in vivo, CSCs were inoculated under the skin to replicate the xenograft model. The tumour weight and tumour growth curve show that after 25 days of intervention, the effect of either lumiflavin or riboflavin on tumours is not obvious, but lumiflavin can be obviously synergistic with DDP in the treatment of tumour, compared to DDP group. Otherwise, riboflavin alleviated the therapeutic effect of DDP on tumour (Figure 3A‐C).

To further explore whether the synergistic anti‐tumour effect of lumiflavin and DDP are related to the changes in mitochondrial function and oxidative stress, several indicators related to oxidative stress, such as GSH‐PX, SOD, and MDA in tumour tissues were tested. The results show that the indicators of tissue antioxidant capacity,^30^ such as GSH‐PX and SOD, were significantly lower in the DDP group than those in the control group (*P* < 0.05 or 0.01), and those in lumiflavin combined with the DDP group were significantly lower than those in the DDP group (*P* < 0.05 or 0.01). Furthermore, MDA, an indicator oxidative damage,^31^ was significantly higher in the DDP group than the control group (*P* < 0.05), and lumiflavin combined with the DDP group significantly higher than that in the DDP group (*P* < 0.05). However, riboflavin weakened the effects of DDP on MAD and SOD in tumour tissue (Figure 3D‐F).

### 3.4 Effects of lumiflavin combined with DDP on apoptosis‐related proteins in a xenograft model

**Figure 4:**
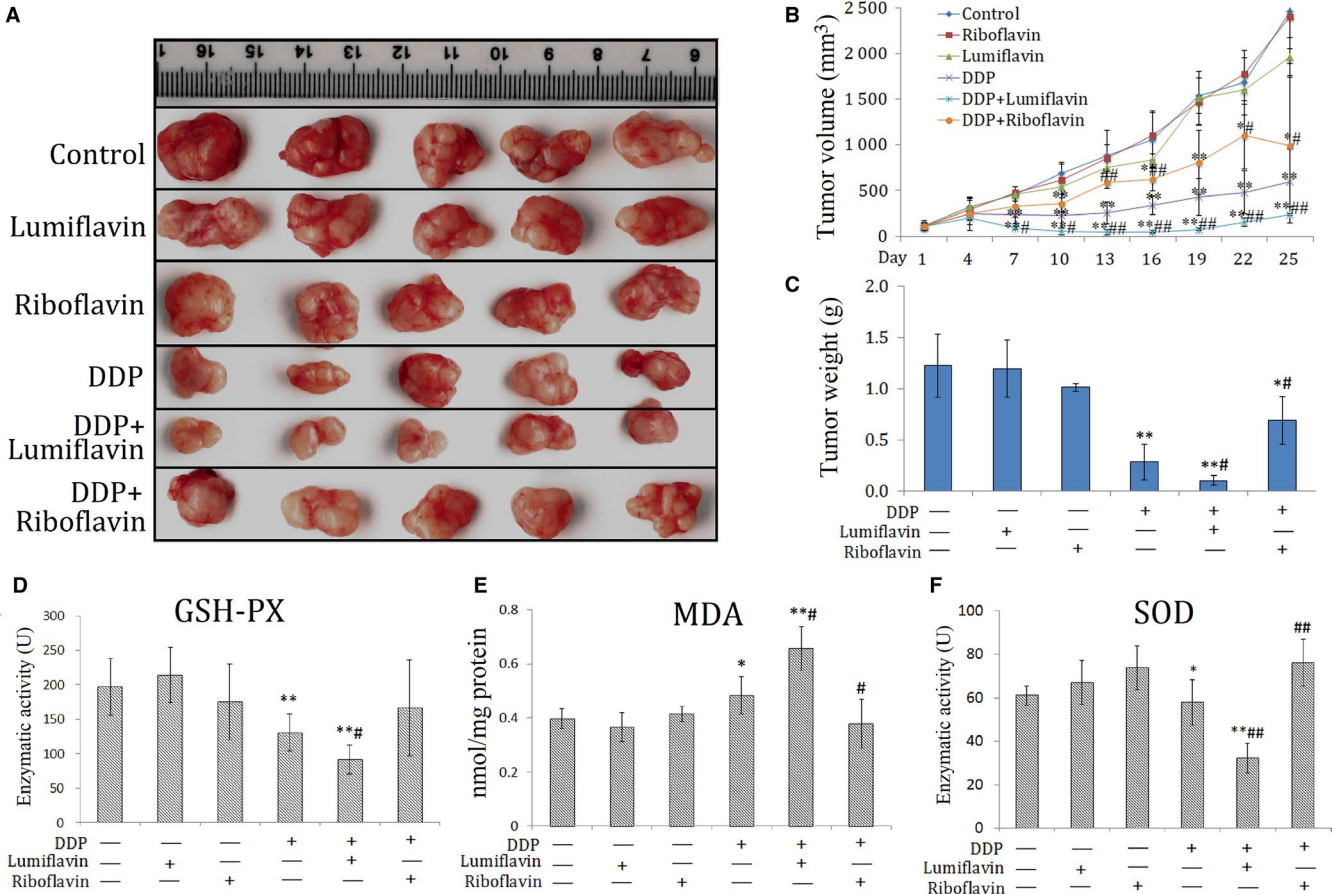
Synergistic effects of lumiflavin and DDP on the mouse xenograft model of ovarian cancer stem‐like cells (CSCs). CSCs of HO8910 (5 × 10^6^) cells were subcutaneously injected under the oxter skin of female BALB/c nude mice. When the tumours reached palpable size (100 mm^3^), mice were intraperitoneally injected for 25 days with saline control, riboflavin (4 mg/kg, once a day), lumiflavin (8 mg/kg, once a day), DDP (5 mg/kg, once a week), DDP + lumiflavin and DDP + riboflavin. Tumour sizes and weight were measured at the end of the experiment. Enzyme activities of GSH‐PX and superoxide dismutase (SOD) and the malondialdehyde (MDA) contents were tested using the corresponding kits, following the manufacturer's instructions. A, Photograph of tumours. B, Tumour growth curve of nude mice. C, Statistical graph of tumour weight. D, Statistical graph of enzyme activity of GSH‐PX. E, Statistical graph of MDA contents in tumour. F, Statistical graph of enzyme activity of SOD. *P < 0.05 compared to control group; **P < 0.01 compared to control group. ^#^ *P* < 0.05 compared to DDP group; ^##^ *P* < 0.01 compared to DDP group. Mean ± SD (n = 5)

To further explore the mechanism of action of lumiflavin and DDP, apoptosis‐related proteins, such as cleaved caspase 3, Bax, B‐cell lymphoma‐2 (BCL‐2) and Bas were detected using Western blot analysis. The results show that the expression of caspase 3 and the apoptotic marker proteins (Bax and Bas) could be increased by DDP (compared to DDP group, *P* < 0.05 or 0.01), and lumiflavin could exacerbate this phenomenon (compared to DDP group, *P* < 0.05). Furthermore, riboflavin could decrease the expression of Bax under DDP treatment and increase the expression of BCL‐2, a protein that inhibits apoptosis.^32^ (compared to DDP group, *P* < 0.05 or 0.01) (Figure 4A,B).

### 3.5 Competitively antagonized content of riboflavin in tumour stem cells in vivo by lumiflavin

**Figure 5:**
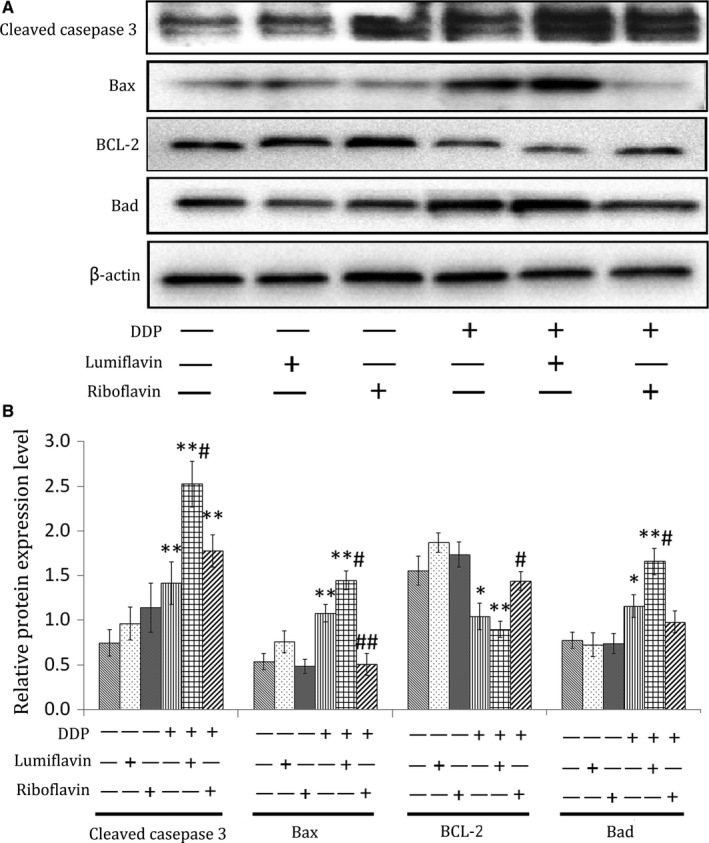
Synergistic effects of lumiflavin and DDP on the down‐regulation of BCL‐2 expression and up‐regulation of the cleaved caspase 3, Bax, and Bad expression in the tumour cells of mice with ovarian cancer stem‐like cells (CSCs) by using the xenograft model. CSCs of HO8910 (5 × 10^6^) cells were subcutaneously injected under the oxter skin of female BALB/c nude mice. When the tumours reached a palpable size (100 mm^3^), mice were intraperitoneally injected for 25 days with saline control, riboflavin (4 mg/kg, once a day), lumiflavin (8 mg/kg, once a day), DDP (5 mg/kg, once a week), DDP + lumiflavin and DDP + riboflavin. Protein expression of the cleaved caspase 3, Bax, Bad, and BCL‐2 in tumour tissues were analysed through Western blot analysis. A, Electrophoretograms of cleaved caspase 3, Bax, Bad and BCL‐2 proteins. B, Graph of relative protein expression as determined by Western blot analysis. ^*^ *P* < 0.05 compared to control group; **P < 0.01 compared to control group. ^#^ *P* < 0.05 compared to DDP group; ^##^ *P* < 0.01 compared to DDP group. Mean ± SD (n = 5)

To determine the correlation between lumiflavin and riboflavin in vivo, the content of riboflavin in tumour tissues was detected through UPLC‐MS/MS The results of the cell experiment show that lumiflavin competitively inhibited the riboflavin content on the DDP treatment group compared to the DDP group (*P* < 0.01). Furthermore, in tumour tissues, the riboflavin content in the groups of lumiflavin combined with DDP is lower than that in the control and DDP group (*P* < 0.01) as shown in Figure 5A,B.

## 4 DISCUSSION

**Figure 6:**
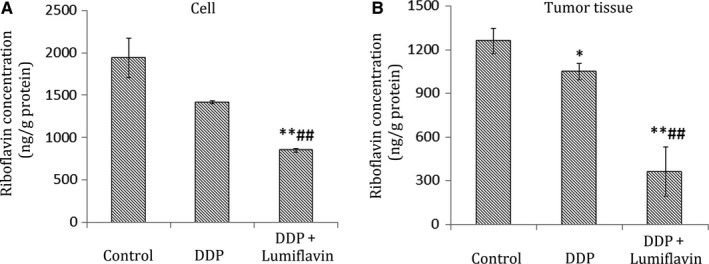
Synergistic effects of lumiflavin and DDP on ovarian cancer CSCs involved in riboflavin. CSCs were seeded in 6‐well plates (1 × 10^6^ cells per well), and then treated for 48 hours as follows: control group, DDP (20 μmol/L) group, and DDP + lumiflavin (20 μmol/L) group. CSCs were subcutaneously injected under the oxter skin of female BALB/c nude mice. Mice were intraperitoneally injected after 25 days with saline control, DDP (5 mg/kg, once a week) and DDP + lumiflavin (8 mg/kg, once a day). The contents of riboflavin in CSCs and tumour tissues were measured using ultra‐high performance liquid chromatography‐tandem mass spectrometry (A) Statistical figure of the CSCs contents of riboflavin. B, Statistical figure of tumour tissue contents of riboflavin. *P < 0.05 compared to control group; **P < 0.01 compared to control group. ^##^ *P* < 0.01 compared to DDP group. Mean ± SD (n = 5)

The cellular function and characteristics of CSCs are closely related to those of riboflavin. Bela Ozsvari et al reported that diphenyleneiodonium, an inhibitor of riboflavin, can eliminate CSCs in tumours and promote cells in a state of metabolic‐quiescence or ‘suspended animation’.^33^ In this study, we examined the riboflavin content and the gene and protein expression of RFT2, which is the main riboflavin transporter; it is the central core during the transfer process of riboflavin.^17^, ^34^ The mRNA and protein expression of RFT2 in CSCs was significantly higher than in NON‐CSCs. Moreover, the riboflavin content in CSCs is more than thrice of that in NON‐CSCs. These results suggest the significant difference in the metabolic capacity of riboflavin between CSCs and NON‐CSCs (Figures 2A‐H and 6). A further study shows that the sensitivity of CSCs to chemotherapeutic drugs was lower than that in NON‐CSCs. DDP and lumiflavin exhibit a synergistic effect on CSCs in a dose‐dependent manner, whereas the effect on NON‐CSCs was not obvious. More importantly, riboflavin significantly reduced the effect of DDP on CSCs, but the effect was not apparent in NON‐CSCs (Figure 1I‐J). Hence, riboflavin was closely related to the sensitivity of CSCs to chemotherapy.

The effect of DDP on tumour cells is closely related to mitochondrial function. The concentration of DDP in mitochondrial DNA (mtDNA) is higher than in nuclear DNA (nDNA), and its clearance in mtDNA is significantly later than in the nDNA, which can induce the metabolic disorder of energy and produce free radicals that damage the cells.^35^ The effects of lumiflavin on the mitochondrial function of CSCs were further discussed, and the changes in mitochondrial function were analysed. The results of cell ROS and mitochondrial membrane potential show that synergistic effect of lumiflavin and DDP on decreasing the mitochondrial membrane potential and the cell content of ROS. Therefore, the effect of lumiflavin is related to the enhanced mitochondrial damage in DDP. Moreover, riboflavin attenuates mitochondrial damage caused by DDP, which agree with previous reports.^36^, ^37^ Hence, we assumed that riboflavin could interfere with the anti‐tumour effect, and lumiflavin enhanced the anti‐tumour effect of DDP through competitive antagonism to riboflavin in mitochondrial function.

Mitochondrial damage can induce apoptosis and decrease the proliferation of CSCs. In this study, the apoptosis rate and cloning abilities of CSCs were detected. Lumiflavin treatment did not affect the cloning efficiency and apoptosis rate of CSCs. However, lumiflavin treatment combined with DDP exerted obvious synergistic effects on decreasing clone formation ability and increasing apoptosis rate of the cells. Moreover, riboflavin could decrease the apoptosis rate in the control or DDP group. These findings suggest that the effects of riboflavin are very important on the apoptosis rate and proliferation of CSCs.

We replicated a xenograft model of CSCs in vitro and administered lumiflavin and DDP in combination or individually to observe the tumour growth curve and tumour weight. The results show that the tumour growth curve in the DDP intervention group was slower than the control group, and the combined intervention of the lumiflavin and DDP group was lower than DDP group. The final tumour weight shows that the combined intervention of lumiflavin and DDP group was lighter than that in DDP group.

Mitochondrial damage is the key event in cell apoptosis. DDP damages the mitochondria, consuming antioxidant enzymes, such as GSH‐PX and SOD, and triggers the irreversible process of apoptosis.^38^, ^39^ It promotes MDA accumulation, which strengthens cell mitochondrial damage.^40^ Riboflavin is very important for maintaining mitochondrial function, while lumiflavin can inhibit riboflavin uptake and suppress the conversion of riboflavin to FAD.^41^, ^42^ In the present study, lumiflavin assisted DDP to reduce the potential of the mitochondrial transmembrane, increase the MDA content, and decrease the enzyme activity of GSH‐PX and SOD in CSCs. Further research shows the synergistic effect of lumiflavin and DDP on increasing the expression levels of apoptosis‐related proteins such as cleaving caspase 3, Bax and Bad in tumour tissues. BCL‐2 can inhibit apoptosis, reduce the production of oxygen‐free radicals and the formation of lipid peroxides, and increase intracellular antioxidants, such as GSH‐PX and SOD.^43^ In this study, the combination of lumiflavin and DDP reduced the expression of BCL‐2 in tumour tissue. The results suggest that lumiflavin interfered with the mitochondrial function and increased tumour apoptosis which was induced by DDP.

Finally, determine whether the synergistic effect of lumiflavin and DDP is associated with the antagonistic riboflavin, the contents of riboflavin in CSCs and tumour tissues were tested. The results show that lumiflavin effectively reduced the level of riboflavin in CSCs and tumour tissues in vitro and in vivo. These findings suggest that the effect of lumiflavin is associated with the reduced riboflavin levels in cells and tissues.

In summary, this study systematically studied the enrichment effect of riboflavin on ovarian cancer CSCs, which is among the important features on chemotherapy sensitivity of CSCs. Lumiflavin can significantly combine with DDP to damage the mitochondrial function, induce the apoptosis and decrease proliferation of CSCs by interfering with the metabolic process of riboflavin. This study not only indicated the possible development of lumiflavin as a chemotherapy sensitizer but also elaborated that interfering with the riboflavin metabolic pathway may be an important target for CSCs. It is an alternative for the treatment of ovarian cancer and other malignant tumours. However, this study has the following limitations: (a) The data do not fully reflect the actual situation of human, because immunodeficient mice were used in this study; (b) The effect of lumiflavin on the phenotypic transformation of CSCs was not studied; (c) The effect of lumiflavin on the multidrug resistance of ovarian cancer cell strains of CSCs was not studied; and (d) Although the results of the preliminary study on the effect of lumiflavin on cells were not significant, systematic toxicological experiments were not studied in vitro and in vivo. The detailed mechanisms of lumiflavin through the riboflavin metabolic pathway on CSCs will be detailed in future studies.

## CONFLICT OF INTEREST

The authors confirm that there is no conflict of interest.

## AUTHOR CONTRIBUTIONS

R.Y. and Z.W. designed the experiments. Z.W. performed the majority of the experiment with the help of R.Y. R.Y. performed the microarray data analysis. R.Y. and S.W. wrote the manuscript.
